# Antimicrobial Properties of Silver Cations Substituted to Faujasite Mineral

**DOI:** 10.3390/nano7090240

**Published:** 2017-08-27

**Authors:** Roman J. Jędrzejczyk, Katarzyna Turnau, Przemysław J. Jodłowski, Damian K. Chlebda, Tomasz Łojewski, Joanna Łojewska

**Affiliations:** 1 Malopolska Centre of Biotechnology, Jagiellonian University, Gronostajowa 7A, 30-387 Kraków, Poland; 2 Institute of the Environmental Sciences, Jagiellonian University, Gronostajowa 7, 30-387 Kraków, Poland; katarzyna.turnau@uj.edu.pl; 3 Faculty of Chemical Engineering and Technology, Cracow University of Technology, Warszawska 24, 31-155 Kraków, Poland; jodlowski@chemia.pk.edu.pl; 4 Faculty of Chemistry, Jagiellonian University, Ingardena 3, 30-060 Kraków, Poland; damian.chlebda@uj.edu.pl (D.K.C.); lojewska@chemia.uj.edu.pl (J.Ł.); 5 Faculty of Materials Science and Ceramics, AGH University of Science and Technology, al. Mickiewicza 30, 30-059 Kraków, Poland; lojewski@agh.edu.pl

**Keywords:** zeolites, silver nanoparticles, paper, antimicrobial

## Abstract

A goal of our study was to find an alternative to nano-silver-based antimicrobial materials which would contain active silver immobilized in a solid matrix that prevents its migration into the surrounding environment. In this study, we investigated whether silver cations dispersed in an atomic form and trapped in an ion-exchanged zeolite show comparable antimicrobial activity to silver nanoparticles (NPs). The biocidal active material was prepared from the sodium form of faujasite type zeolite in two steps: (1) exchange with silver cations, (2) removal of the external silver oxide NPs by elution with Na_2_EDTA solution. The modified biocidal zeolite was then added to paper pulp to obtain sheets. The zeolite paper samples and reference samples containing silver NPs were tested in terms of biocidal activity against an array of fungi and bacteria strains, including *Escherichia coli*, *Serratia marcescens*, *Bacillus subtilis*, *Bacillus megaterium*, *Trichoderma viride*, *Chaetomium globosum*, *Aspergillus niger*, *Cladosporium cladosporioides*, and *Mortierella alpina*. The paper with the modified faujasite additive showed higher or similar antibacterial and antifungal activities towards the majority of tested microbes in comparison with the silver NP-filled paper. A reverse effect was observed for the *Mortierella alpina* strain.

## 1. Introduction

Since ancient times, the antimicrobial activity of silver has been widely recognized. Silver can act against a variety of organisms including bacteria, viruses, and fungi with rare cases of resistance [1,2,3]. Due to silver’s outstanding properties and also because of the development of nanotechnology, the application of silver nanoparticles in different areas of industry (e.g., pharmaceutical and clothing) has shown a growing trend. However, an awareness of the risks of silver NP contamination in the environment and in human beings has recently been awakened [4,5]. For example, it has been reported in References [6,7] that silver NPs damage mitochondria in cells of not only bacteria, but also other organisms, including mammals. In all, the effects of their release into the ecosphere are hard to predict.

It seems that studying a simpler system, such as that of pure Ag NPs, may provide some hints to elucidate the zeolite biocidal properties of silver. Even though silver NPs have been studied in terms of their antimicrobial activity for over 20 years, there are still a great many controversies concerning their influence on bacteria and fungi. What is known is that the biocidal mechanism of silver NPs assumes silver-induced oxidation of disulfide or sulfhydryl groups of proteins that cause metabolic disorders and, in consequence, microorganism death [8,9]. Accumulation of silver nanoparticles in microbial membranes has been shown to cause increased permeability [10], and finally membrane damage by free radicals that are formed as an effect of the presence of the NPs [11]. Silver cations are also able to enter bacterial cells and to chelate DNA [9,12]. Much less is known concerning their antifungal mechanisms. 

The main problem encountered in studying silver and other NPs is that their properties depend on their size and distribution, which in turn determines their bioactivity [13,14]. Moreover, it seems difficult to distinguish nanoparticle-specific effects from ionic effects which often occur simultaneously. In general, NPs are efficient in forming metal ions of known antimicrobial properties [15], although NPs can also be a source of Ag ions, as recently reviewed by Sheehy et al. [16]. The results published to date concerning NP toxicity have not paid attention to the presence and concentration of Ag^+^, which can increase NPs’ size with time and with the accessibility of oxygen. Thus, it can be inferred that the antibacterial properties of NPs are not due to their specific properties, but to the cations that can be formed under specific conditions [17]. The study of the effects of NPs becomes even more complex if we take into account that NPs have a tendency to agglomerate, e.g., in nutrient broth, and thus they are unsuitable for other assays apart from disk diffusion. 

The main question which is stated in this paper is whether commonly used silver NPs can be replaced by other silver-containing materials where silver is immobilized, but shows antimicrobial activity comparable to the NPs. The idea is not only to introduce silver to a solid matrix, but also create strong bonds to protect against the silver’s release from the material. A simple solution may be the application of zeolites, to which silver cations can be introduced by ionic exchange. No matter the mechanism of silver NPs’ biocidal action, we assume that both the cations and NPs can easily migrate to surroundings if they are not immobilized by certain means. To minimize the risk of interactions with cells of other organisms [18], the stable binding of Ag forms into structural matrices in the form of, e.g., zeolites are considered [19,20,21]. Zeolites are aluminosilicates, unique in that their crystal lattice possesses both channels and voids of a size of less than 1 nm. Due to unsaturation of the coordination number, the acidic OH groups in Al cations present in zeolite voids can be moderately easily exchanged by other cations. It has to be pointed out, however, that the procedure of ion exchange from solutions does not secure the immobilization of silver cations, because external, loosely attached silver oxide NPs or crystallites also form on the surface of zeolites [22]. For this reason, the zeolite exchanged with silver requires more treatment to remove any loosely bound or unbound silver forms. 

Another question that arises concerning the application of silver exchanged zeolite materials, is what would be the mechanism of their antimicrobial action if silver is trapped within the zeolite lattice? According to the pioneering studies of antibacterial activity of silver ion-exchanged zeolites, the mechanisms involve the formation of a water film over the zeolite and the exchange of Na to Ag or other cations (as well as other types of silver bonding, e.g., AgCl). 

The main objective of this study is to evaluate the antimicrobial and biostatic properties of recently patented faujasite containing immobilized silver cations [23], used as a paper additive, and to compare it with its silver NPs counterpart. Another objective is to prove the stability of silver in the materials by showing that it is not released to the surrounding environment.

## 2. Materials and Methods

### 2.1. Zeolite Preparation

Y-type zeolite (faujasite, FAU) in the form of powder (surface area: 900 m^2^/g) was purchased from Zeolyst International (CBV-100, Zeolyst C.V., Oosterhorn, Holland). The zeolite in its sodium form (13 wt %) was used further for the ion exchange after its conditioning at 23.5 °C and 50% RH (relative humidity) for 12 h. Silver nitrate was obtained from Avantor Performance Material Company (Gliwice, Poland) and disodium ethylenediaminetetraacetate dihydrate (Na_2_EDTA) was from Sigma-Aldrich (Saint Louis, MO, USA).

In order to remove the unexchanged (non-bonded) silver from the external surfaces of zeolite, the prepared samples were washed by: (1) portions of 10 cm^3^ of 0.01 M Na_2_EDTA solution, and (2) 10 cm^3^ of deionized water (the procedure was repeated five times). The washing procedure was optimized in terms of EDTA concentration, the volume of the solution, and the number of repetitions. To achieve this, the 0.01 M EDTA solution was subsequently added in portions until the silver cations concentration in the eluent reached minimum constant value, which was monitored by XRF (X-Ray Fluorescence) spectroscopy. As a reference, the samples were also washed by the equivalent portions of water. After each step, the zeolite suspension was centrifuged (4000 rpm) and then dried according to the same conditions given below. 

Zeolite exchanged with silver was used as an additive to paper pulp (44 wt %). The zeolite samples were prepared by suspending 1.00 g of zeolites in 100 cm^3^ of 0.1 M AgNO_3_ solution in deionized water. Due to the light sensitivity of silver, suspensions were stirred in darkness (300 rpm, 1 h). After ion exchange, the samples were filtered and washed with deionized water. After preparation, the obtained samples were dried in an oven at 60 °C for 8 h.

### 2.2. Paper Preparation

Paper sheets were prepared from cellulose (Whatman filter paper—Maidstone, UK) and silver-modified faujasite. Prior to sample formation, the paper sheets were conditioned according to the ASTM D685 norm [24]. 

Whatman filter papers were cut into 4 cm × 4 cm pieces and then suspended in 400 cm^3^ of deionized water in an autoclave and homogenized (IKA T18 Ultra-Turrax with stainless steel dispersing elements, Staufen im Breisgau, Germany). This allowed us to obtain the suspensions of the paper in deionized water. Just after the formation of the suspension, an appropriate amount of active material was added (as described in 2.1). 

To obtain paper sheets, the pulp was deposited on a custom-build vacuum table. Wet paper sheets (about 15 cm × 20 cm) were dried on the glass surface at ambient temperature. Small circle samples were cut out by hole punching and then sent for microbial tests.

To assess the silver content in the thus-prepared samples, elemental analysis using an XRF spectrometer (Thermo Scientific ED-XRF, Waltham, Massachusetts, MA, USA, thick Cu filter, *K*_α_ = 22.36 eV) was carried out, which was preceded with sample digestion in boiling 65% nitric acid (Sigma Aldrich, Saint Louis, MO, USA) for 15 min. The silver determination was performed according to an external standard method.

### 2.3. Reference Materials Preparation

The reference paper samples containing silver NPs were also prepared. Silver nanoparticles were obtained by sonication (10 min) of AgNO_3_ (Sigma Aldrich, Saint Louis, MO, USA) solution (0.1 M) containing a low amount of ethanol (1.5 vol %)—the beaker was placed in an ice bath to maintain a temperature of reaction below 60 °C. Prior to the sonication, the thus-prepared suspensions were purged with an inert gas (Ar, Linde Gaz Polska, Kraków, Poland) for 60 min, after which an ultrasound frequency of 20 kHz was applied (the average power of the ultrasound was equal to 90 W). The pulsating sonication program was set up. The sonication to downtime ratio was set to 3:1 (min). 

### 2.4. Antimicrobial Tests

*Escherichia coli*, *Serratia marcescens*, *Bacillus subtilis*, *Bacillus megaterium*, *Trichoderma viride*, *Chaetomium globosum*, *Aspergillus niger*, *Cladosporium cladosporioides*, and *Mortierella alpina* from the culture collection of the Laboratory of Plant-Microbial Interaction Group of the Jagiellonian University (Krakow, Poland) were selected for the laboratory tests on the basis of pilot studies and the ability of these organisms to grow on paper or on/in materials such as plants, food, etc. in which they could be packed. In the case of bacteria, uniform samples of bacteria in saline solution were applied to the surface of agar (NA, Difco Laboratories, Detroit, MI, USA) and spread with a disposable spreader. The bacteria were pre-grown at 32 °C for two days. In each Petri dish, a single disc of paper (5 mm in diameter) was placed. The culture was grown for another three days at 32 °C, and subsequently the temperature was decreased to 25 °C for three days. For every strain and each paper sample, five repetitions were applied. The growth of microorganisms was monitored on a daily basis. Finally, each of the Petri dishes was opened in a laminar chamber. The paper sample was lifted using sterile forceps and turned upside-down, exposing a part that was previously adhered to the agar with bacteria. To quantify the bacterial population, the LuciPac Pen test (ATP + AMP, Hygiene Monitoring test kit from Kikkoman Corp., code 60331, Noda, Japan) was used. Swabs were set perpendicular to the disc in the central part, and the tip was turned 360 degrees. The relative content of ATP and AMP (RLU—relative luminescence units) was evaluated with a lumitester. 

In the case of fungi, the paper discs (three per petri dish) were first placed on standard potato dextrose agar (PDA medium) and fungi in the form of mycelium or spores were introduced at a distance of 2 cm from the discs. For each fungal strain and each type of treatment, the procedure was repeated three times. The cultures were kept in a dark chamber at 27 °C for up to three weeks, depending on fungal growth. Following this period, the inhibition zone around the paper disc was measured and the ATP/AMP test was conducted as described above for the bacteria, with an exception concerning the difference in sampling of the material from the top of the paper discs. 

## 3. Results

The description of the samples used in the microbiological studies together with the silver content is given in Table 1. The silver content in the faujasite samples was optimized and is the minimum value necessary to achieve biocidal effects in paper samples. 

### 3.1. Bacterial Strains

**Figure 1:**
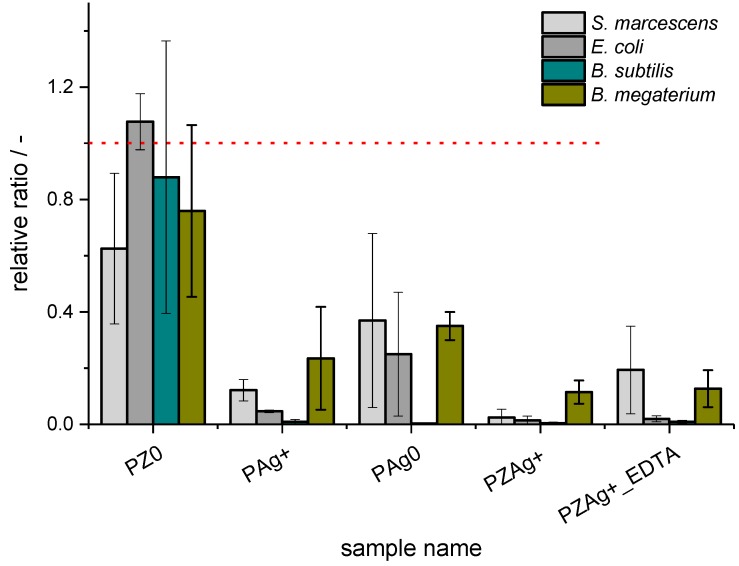
The relative ratio of adenosine phosphates as compared to control values (bacteria).

The paper sample P (Table 1) with no additives served as a gauge to which the other samples were related. For the P sample among the four bacterial strains studied (Figure 1), the highest adenosine phosphate (ATP, ADP, AMP) concentrations were observed in *Bacillus megaterium*. For other bacterial strains, the ATP/AMP concentration was lower. Silver addition in any of the studied forms apparently suppressed the growth of all bacteria in direct contact with the paper samples as compared to the P and PZ0 reference samples. No inhibition zone was visible around the discs, but below the discs (as well as on the top) significantly lower RLU counts were reported. The most profound drop in RLU values was observed for *E. coli* and *B. subtilis*, and a lesser drop was observed for *B. megaterium*. In the case of *Serratia marcescens*, the biocidal effect of silver was moderate and significant only for PZAg+. An important observation is that the activity of the silver NPs-containing sample was at least twice as low as the activity of the zeolite silver-exchanged samples judging by the ATP content. Taking into account all of the treatments and all bacterial strains, the strongest antibacterial effect was seen for PZAg+. The detailed results of the relative content of ATP and AMP for analyzed bacteria are presented in the Supplementary Material (Figures S1–S4).

### 3.2. Fungal Strains

**Figure 2:**
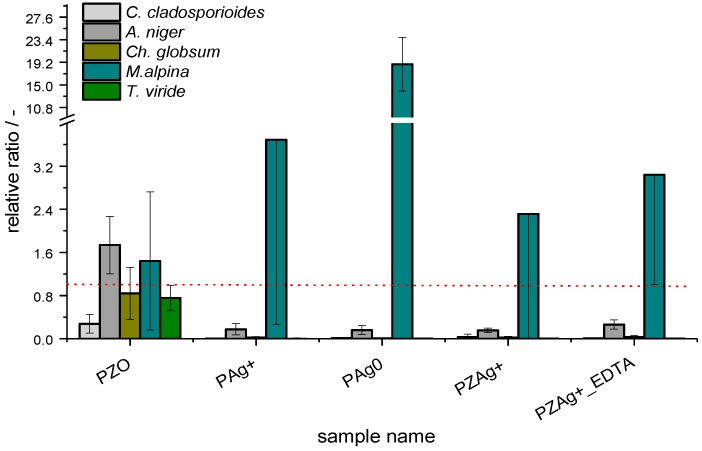
The relative ratio of adenosine phosphates as compared to control values (fungi).

The effects of Ag on fungi (Figure 2) were not as obvious as in the case of bacteria. Fungi differed not only in the rate of growth but also in the appearance of colonies, the abundance, or/and the mode of spore development. A visual comparison of the microbial activity of the samples with silver NPs (PAg0) and the modified zeolite sample (PZAg+_EDTA) with reference to the control paper sample P is presented in Figure 2. No significant differences in the growth rate of *T. viride*, *Ch. Globosum*, or *M. alpina* on P and PZ0 reference samples were noted, except for the decreased growth of *C. cladosporioides* (compared to P) demonstrated only by PZ0. Also in the case of fungi, silver significantly affected their growth (*Ch. globosum*, *C. cladosporioides*, and *A. niger)*. All Ag treatments resulted in a similar inhibition of fungal growth. However, the opposite was shown for *M. alpina*, where most of the Ag treatments resulted in an increase of mean RLU counts by up to 18-fold. More detailed information can be found in the Supplementary Material.

**Figure 3:**
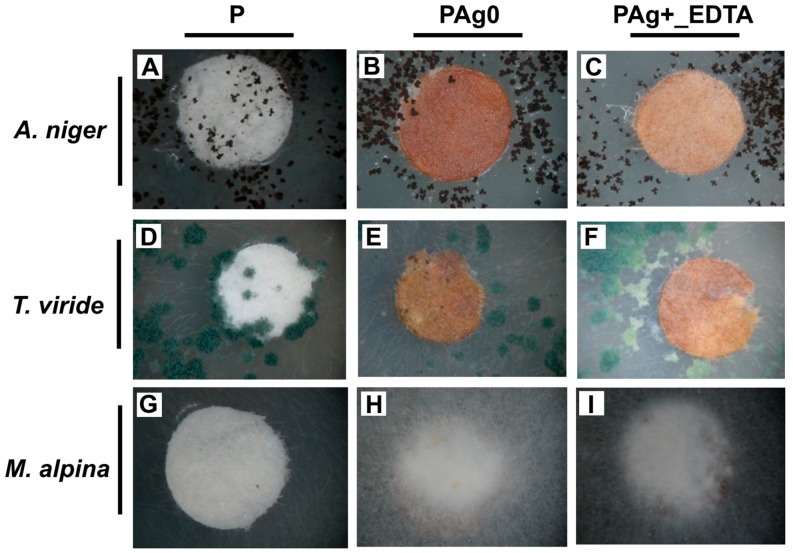
Examples of antifungal activity of silver-exchanged zeolite PZAg+_EDTA (**C**,**F**,**I**); reference samples of pure paper P (**A**,**D**,**G**); and paper with silver nanoparticles PAg0 (**B**,**E**,**H**).

In addition to the general tendency in RLU values, the Ag treatments delayed maturation of spores, fruiting bodies formation in *Ch. Globosum*, and the inhibition of conidiophore production in *A. niger*. What is more, *T. viride* spores exhibited weaker pigmentation. Conidia that landed on the modified paper did not germinate (Figure 3).

As regards the fungi distribution within the samples, the mycelium of *A. niger*, *T. viride*, and *C. cladosporioides* did not penetrate the PZAg^+^_EDTA sample. For *Ch. Globosum*, a poor inhibition zone was visible for PZAg^+^, while in the case of other samples the inhibition zone was quite visible. The mycelium of *Mortierella alpina* easily colonized paper samples enriched with Ag. The detailed results of the relative content of ATP and AMP for analyzed fungi are presented in the Supplementary Material (Figures S5–S9).

## 4. Discussion

The idea of our study was to verify the antimicrobial properties of material that contains silver dispersed on an atomic scale, yet trapped in the material structure so as to prevent the leakage of silver into the surrounding environment. Such material, if it shows sufficient antimicrobial activity, can be regarded as an alternative to silver NPs additives to different materials. In this study, paper was chosen as a carrier for both NPs and the silver-modified faujasite samples. Faujasite exchanged with silver was chosen in this study because it showed the highest biocidal action against *E. coli* amongst the studied samples exchanged with silver (MCM-56, ZSM-5), and because it is commercially easily available.

**Figure 4:**
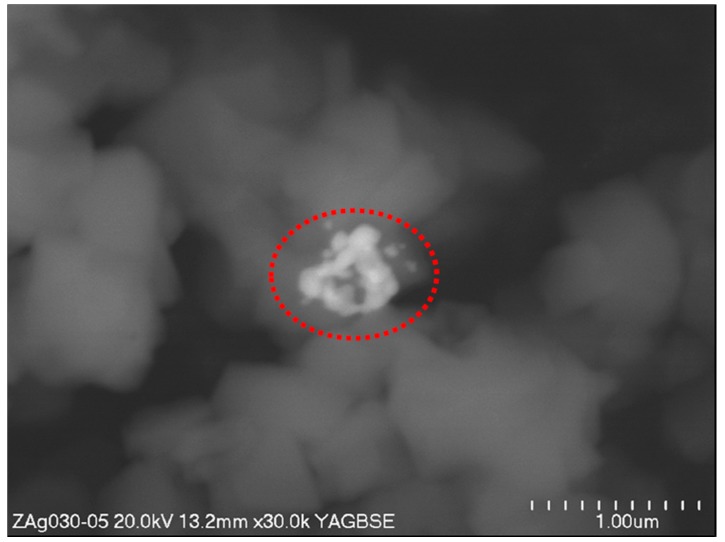
SEM image of faujasite exchanged with silver cations (as ZAg+)—area of interest is marked by red circle.

It is known that, after ion exchange, the zeolites also contain non-bonded metal oxide particles which can be seen in the SEM image taken in view of back-scattered electrons (Figure 4). In contrast to secondary electrons, the scattered electrons are sensitive to the mass of elements on which they are reflected, giving a contrast in the photographs. On the untreated silver-exchanged zeolite there are lighter spots which come from the silver oxide. To remove them from the material, a chelating agent was used. The EDTA sodium salt is a molecule which, due to its big size, is not able to fully penetrate faujasite channels or voids. The fact that, after several repetitions of washing with EDTA, the amount of silver reached low and constant value shows that the non-bonded silver was removed from the zeolite, leaving only the silver cations that are bound to Brønsted acidic sites on Al atoms in the faujasite structure. Indeed, when we consider the structural parameters of faujasite crystal, the pore diameter is 7.4 Å while the voids diameter 12 Å. The EDTA anion in its most condensed octahedral conformation, with the diameter of the basis building the pyramid, reaches 7.5 Å. The lack of non-bonded silver in the studied material was also proven by the lack of a “halo zone” (free of bacterial growth) around the paper discs in the case of bacteria. Bacteria react to silver only when in direct contact with the discs, but the mechanism needs further research.

The luminescence method is commonly employed to monitor microbiological contamination. In this study, it was found useful for evaluating bacterial and fungal growth. The method allowed for an objective, number-wise comparison of microbial growth in the presence or absence of Ag. The adenosine phosphate content can be rapidly depleted in stressed or dying cells [25,26,27,28], but in healthy cells it is reasonably constant and proportional to cellular biomass and changes with microorganism cell size. This might be the cause of the higher adenosine phosphate concentration in *B. megaterium*, which is among the largest known bacteria, while other bacterial species had lower sizes [29] in addition to lower ATP/ADP/AMP concentrations. The differences between bacterial taxa might be the reason for some mistakes in microbe evaluation in the case of environmental samples where the identities of bacteria are unknown. The less drastic effect of Ag presence in case of *B. megaterium* can be due either to its higher tolerance to Ag ions, better adaptation to growth on paper, or simply to different access of the metal to cells within the colony. According to the results, no differences between G-positive and G-negative bacteria were found, although literature data indicates that G-negative bacteria are more tolerant/resistant to heavy metals [30]. Silver has no known beneficial effects on bacterial cells and thus it is toxic even at low concentrations [31]. As reviewed by Bruins et al. [32], the bacterial metal tolerance/resistance results from biochemical and structural properties, physiological and genetic adaptation including morphological changes of cells, as well as environmental modifications of metal speciation [33]. Diverse mechanisms, both chromosomal and plasmid-dependent, are used by bacteria to overcome the presence of Ag in the environment [33,34,35]. These mechanisms protect the organisms from oxygen radicals produced in the Fenton reaction [36], but also determine the ability to precipitate metal phosphates, carbonates, and sulphides. In addition, metals can be detoxified by negatively charged residues of membrane components and exopolymers, energy-dependent metal efflux systems, and intracellular sequestration with low molecular weight cysteine-rich proteins [37]. Similar mechanisms have been shown in fungi subjected to silver cations [38,39,40]. 

The antibacterial effect of Ag NPs has been the subject of significantly more studies [41,42,43,44,45] than their antifungal effect. As found previously [46], Ag NPs stimulated the growth of *Mortierella alpine*, which was similarly tolerant to Ag forms used in the present study. Here its growth rate was up to four times higher compared to the control paper P and PZ0 samples. It is important to remember that some microbes can be tolerant to diverse forms of silver while preparing new products.

In the present study, various forms of Ag were introduced into paper (Table 1). The reference samples were chosen in terms of their known antimicrobial activity (PAg^+^, PAg^0^). According to the available literature, the paper containing silver NPs was expected to have antimicrobial properties, which were studied using simplistic disc assay [47,48]. This method is fast, cheap, and widely used in testing antibiotics and other soluble molecules. It also allowed us to test the mobility of the tested substances. A question to answer was whether the new modified zeolite sample (PZAg^+^_EDTA) with the incorporated silver cations would show biocidal properties comparable to silver NPs. The results presented in Figure 1 and Figure 2 confirm our hypothesis. It should be pointed out that in the literature there are also examples of the results describing antimicrobial properties of silver-exchanged zeolites, but they are limited to the analyses of *Eschericha coli* [49,50,51]. We would like to point out that the zeolite samples used in the cited references were prepared only by ionic exchange and thus contain both silver oxide nanoparticles located on the zeolite external surface as well as silver cations embedded into the zeolite matrix. 

In this study, no inhibition zone surrounding both the Ag NPs-embedded paper PAg0 and the faujasite-containing paper samples PZAg+ and PZAg+_EDTA was formed. In bacterial assays, colony growth was inhibited at the edge of the paper, whereas in fungal assays, an “quasi-inhibition zone” penetrated by individual hyphae forming a loose mycelium of significantly lower density was observed. It needs to be mentioned here that fungal mycelia grow by hyphae elongation, whereas bacterial colonies expand by multidimensional cell division, thus the quasi-inhibition zone was probably formed due to the inhibition of hyphae branching/ramifying elicited by contact with Ag NPs on the surface of the paper. Consequently, the lack of an inhibition zone indicates the lack of mobility of the silver NPs or silver cations from the paper samples. 

## 5. Conclusions

The aim of this study was to obtain and assess the antimicrobial properties of silver-exchanged faujasite with firmly bonded silver, regarded as an alternative to silver NPs. The faujasite mineral was chosen due to its highest biocidal activity compared to other silver-exchanged zeolites (MCM-56, ZSM-5). The methods of preparation were based on the classical ion exchange from silver nitrate solution followed by washing the exchanged zeolite with a solution of sodium salt of EDTA. The elution allowed for the disposal of external unattached silver (oxide or hydroxides), thus leaving only silver cations attached to the acidic OH groups in Al^3+^. 

The results showed similar antiseptic properties of the silver-exchanged faujasite in comparison to the reference samples of silver NPs and pure faujasite added to paper. The high activity was shown for the majority of tested bacterial and fungal strains: *Escherichia coli*, *Serratia marcescens*, *Bacillus subtilis*, *Bacillus megaterium*, *Trichoderma viride*, *Chaetomium globosum*, *Aspergillus niger*, *Cladosporium cladosporioides. M. alpina* was the only strain which was resistant to the biocidal activity of the manufactured material and to the Ag NPs-containing sample. The material inhibited the growth of the tested microorganisms by 90–95%. 

## Figures and Tables

**Table 1:** Description of samples used.

Sample Name	Studied Component	Description	Silver Content, wt %
P	pure cellulose	Whatman filter paper No. 1	0
PZ0	sodium form of faujasite, FAU	pure faujasite suspended in paper	0
PAg+	silver cations, Ag^+^	silver nitrate dissolved in paper	0.5 ± 0.1
PAg0	silver nanoparticles, Ag^0^	silver nitrate sonicated and suspended in paper	0.3 ± 0.1
PZAg+	silver cation-exchanged faujasite and silver oxide nanoparticles, AgFAU, Ag_2_O	the exchanged faujasite suspended in paper	1.5 ± 0.1
PZAg+_EDTA	silver cation-exchanged faujasite, AgFAU	the exchanged faujasite washed with Na_2_EDTA and then suspended in paper	1.1 ± 0.1

## Supplementary Materials

The following are available online at www.mdpi.com/2079-4991/7/9/240/s1, Figure S1: *Serratia marcescens;* Figure S2: *Escherichia coli*; Figure S3: *Bacillus subtilis*; Figure S4: *Bacillus megaterium*; Figure S5: *Aspergillus niger*; Figure S6: *Trichoderma virdi*; Figure S7: *Cladosporium cladosporioides*; Figure S8: *Chaetomium globosum*; Figure S9: *Mortierella alpine*.
