# Supplementary material for: Antimicrobial Properties of Silver Cations Substituted to Faujasite Mineral
Source: Nanomaterials (Basel). 2017 Aug 27;7(9):240. doi: 10.3390/nano7090240 (PMC5618351; doi:10.3390/nano7090240)
Supplement: Supplementary file 1 [file nanomaterials-07-00240-s001.pdf]

nanomaterials

Supplementary Materials

Antimicrobial properties of silver cations substituted to faujasite mineral

Roman J. Jędrzejczyk [1,*], Katarzyna Turnau [2], Przemysław J. Jodłowski [3], Damian K. Chlebda [4], Tomasz Łojewski [5] and Joanna Łojewska [4]

[1] Malopolska Centre of Biotechnology, Jagiellonian University, Gronostajowa 7A, 30-387 Kraków, Poland
[2] Institute of the Environmental Sciences, Jagiellonian University, Gronostajowa 7, 30-387 Kraków, Poland; katarzyna.turnau@uj.edu.pl
[3] Faculty of Chemical Engineering and Technology, Cracow University of Technology, Warszawska 24, 31-155 Kraków, Poland; jodlowski@chemia.pk.edu.pl
[4] Faculty of Chemistry, Jagiellonian University, Ingardena 3, 30-060 Kraków, Poland; damian.chlebda@uj.edu.pl (D.K.C.); lojewska@chemia.uj.edu.pl (J.Ł.)
[5] Faculty of Materials Science and Ceramics, AGH University of Science and Technology, al. Mickiewicza 30, 30-059 Kraków, Poland; lojewski@agh.edu.pl
* Correspondence: roman.jedrzejczyk@uj.edu.pl; Tel.: +48-12-664-6117

The relative content of ATP and AMP (expressed in the RLU - relative luminescence units) of selected fungi and bacteria for analysed materials (A) and their distribution within the samples (B).

The petri dishes which correspond to the reported new material (PZAg+_EDTA) were indicated by the “red dot” signs.

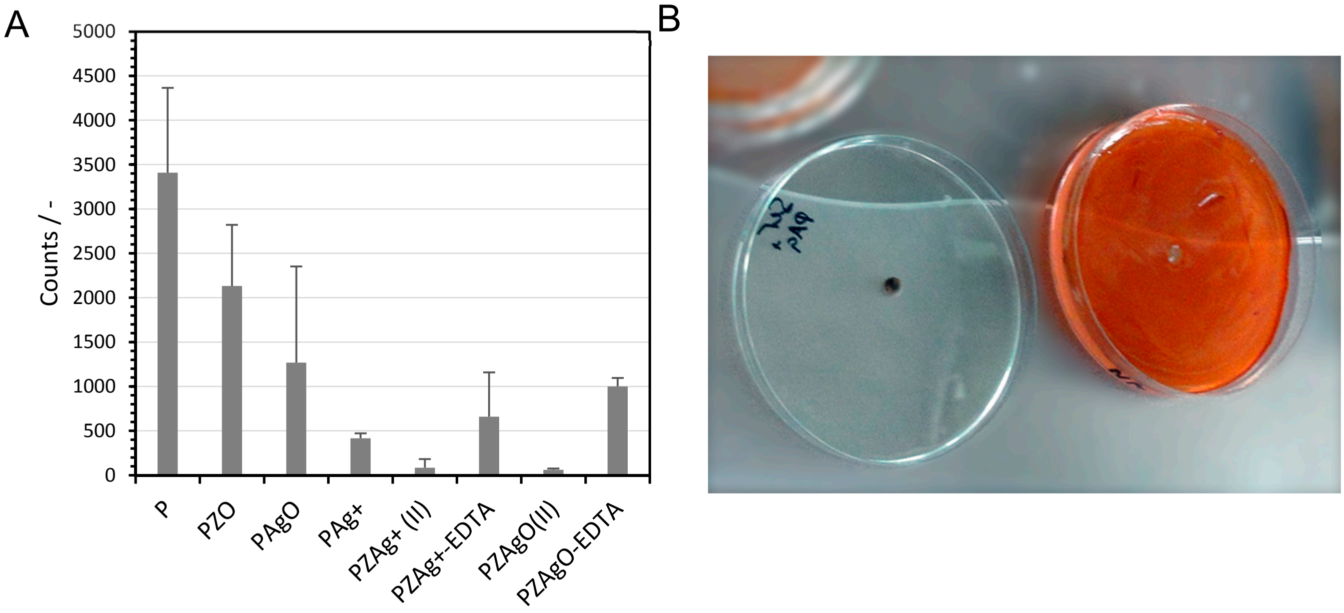

Figure S1 *Serratia marcescens*

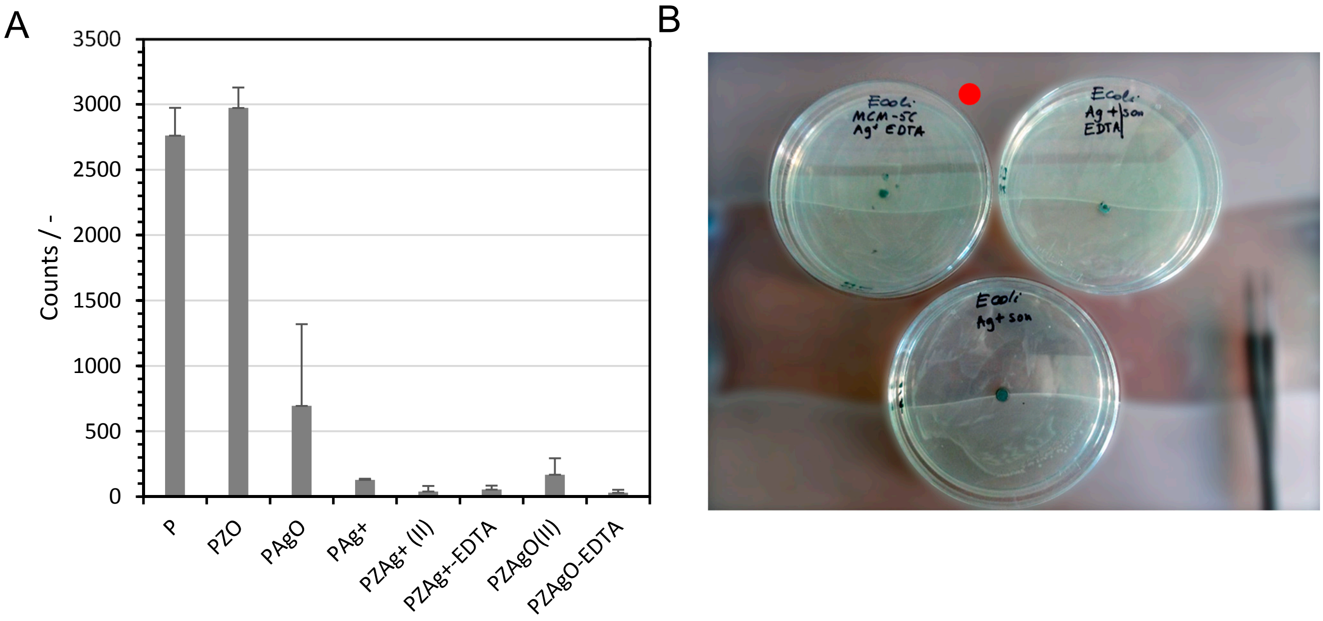

Figure S2 *Escherichia coli*

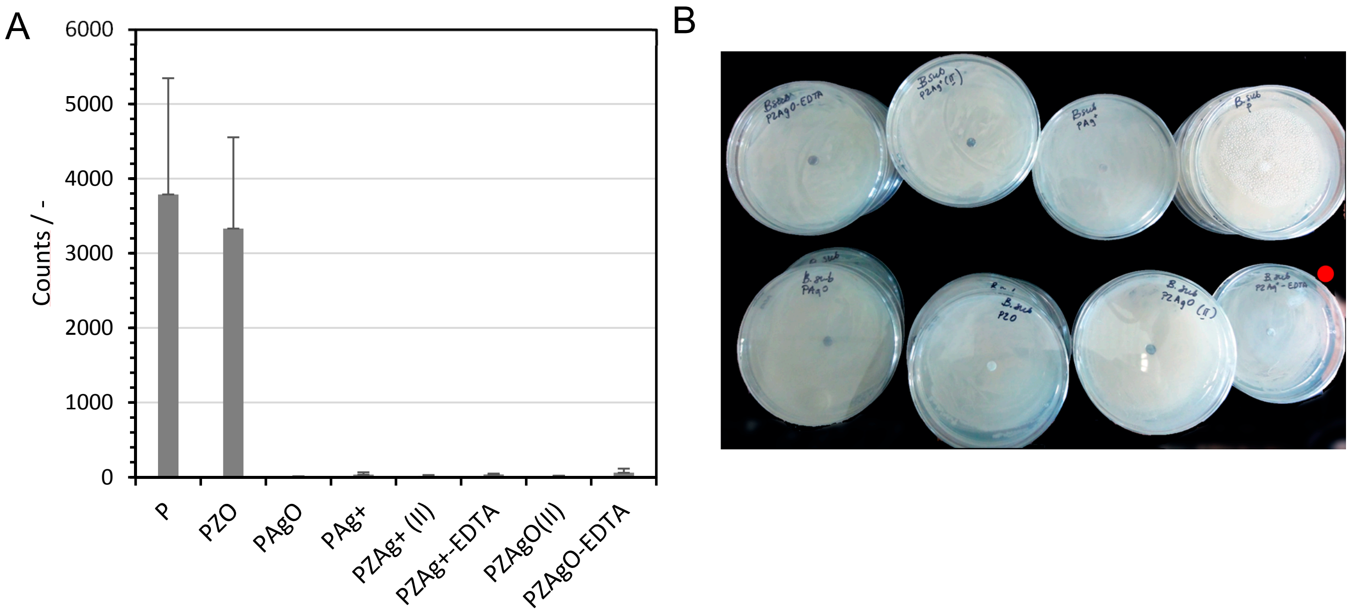

Figure S3 *Bacillus subtilis*

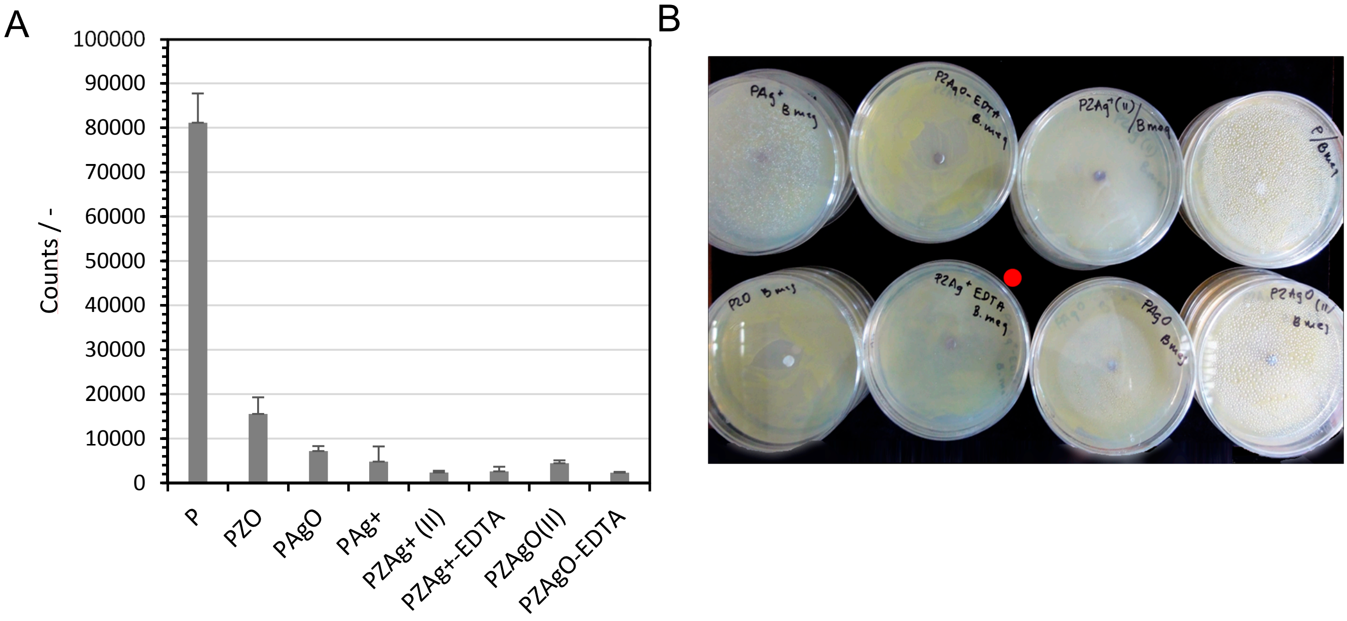

Figure S4 *Bacillus megaterium*

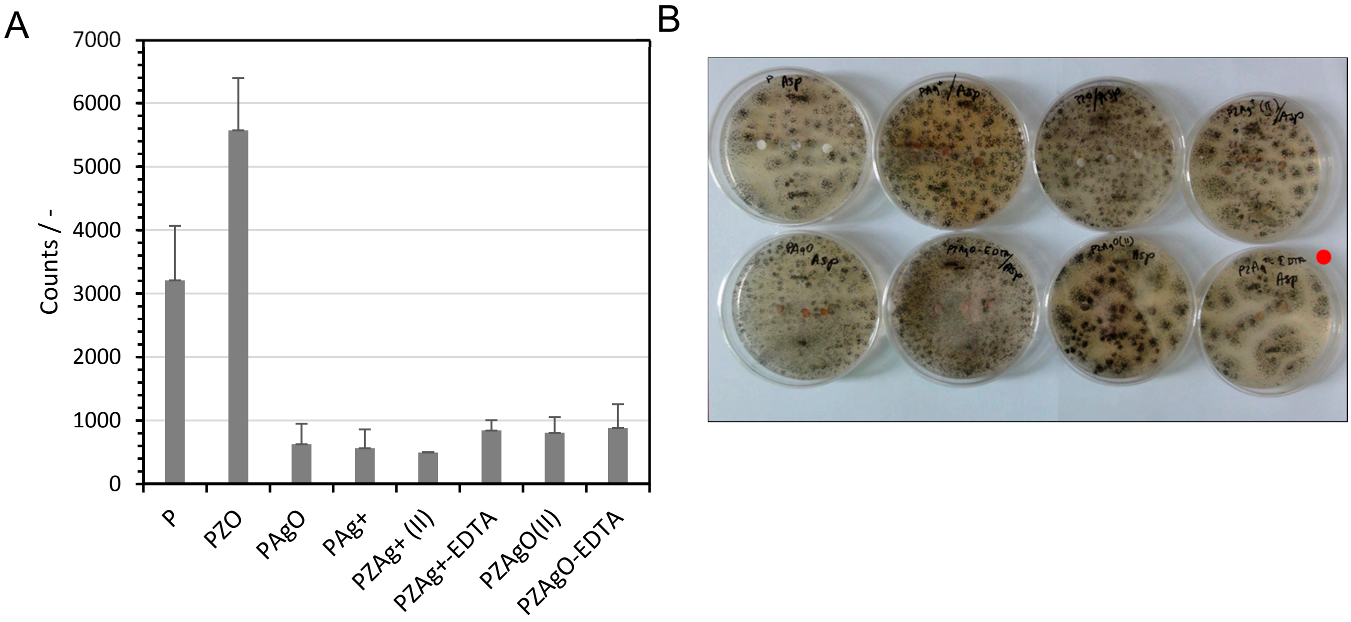

Figure S5 *Aspergillus niger*

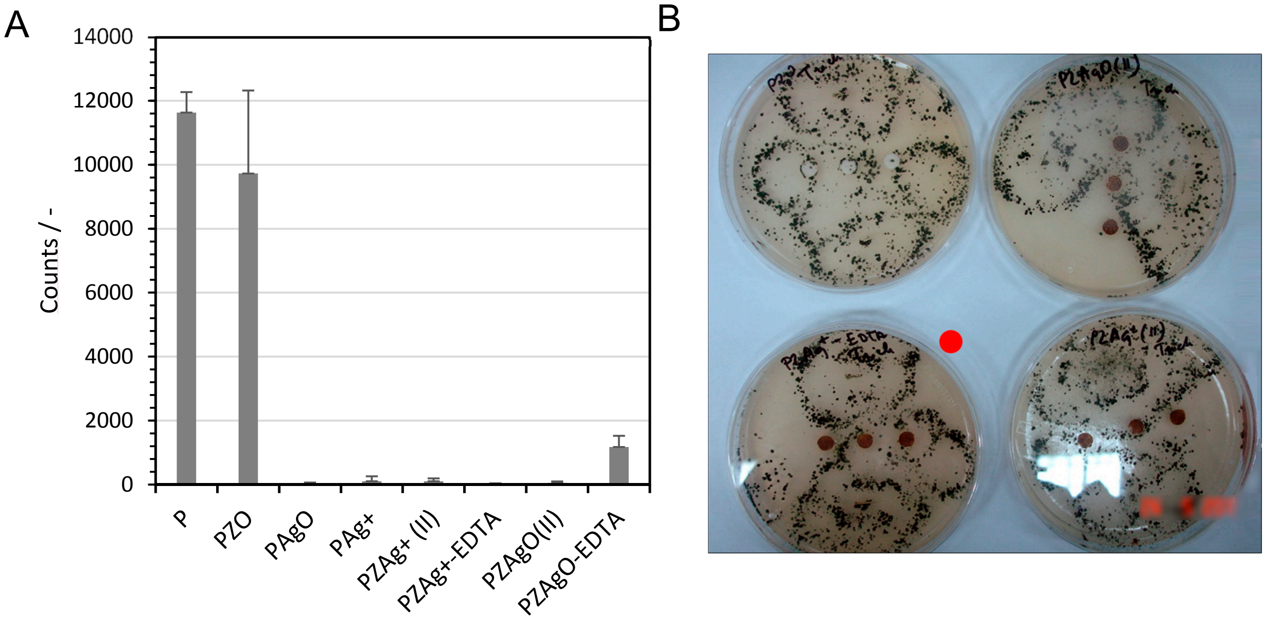

Figure S6 *Trichoderma virdi*

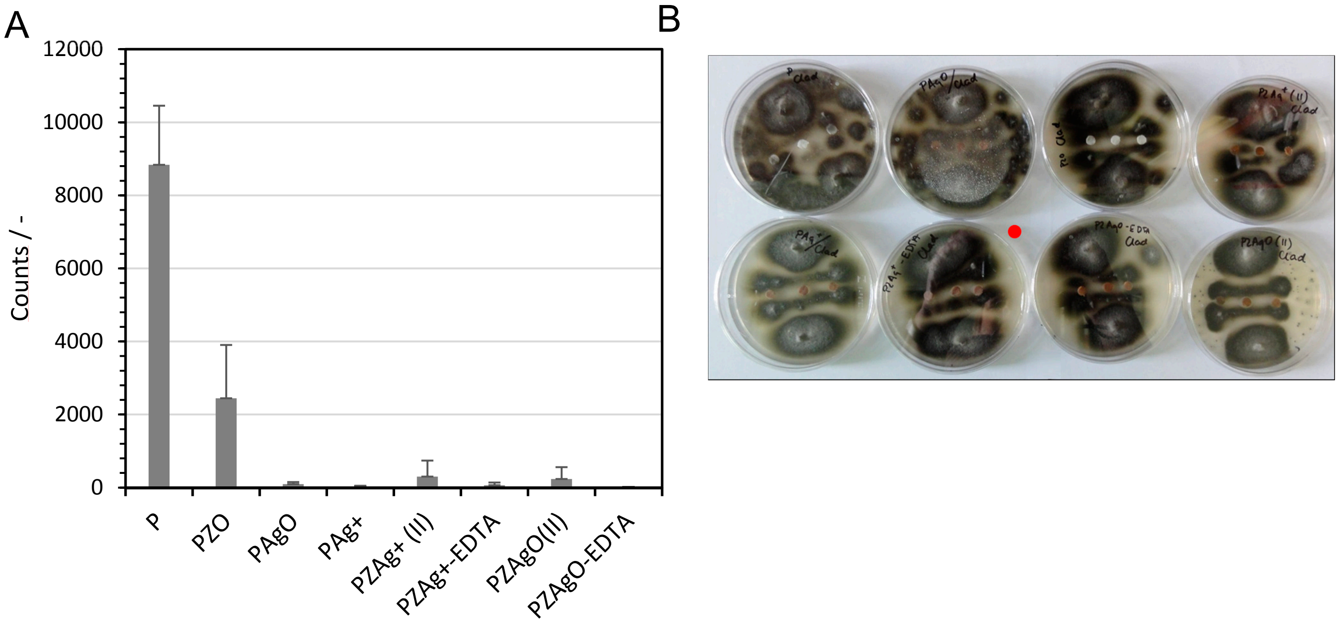

Figure S7 *Cladosporium cladosporioides*

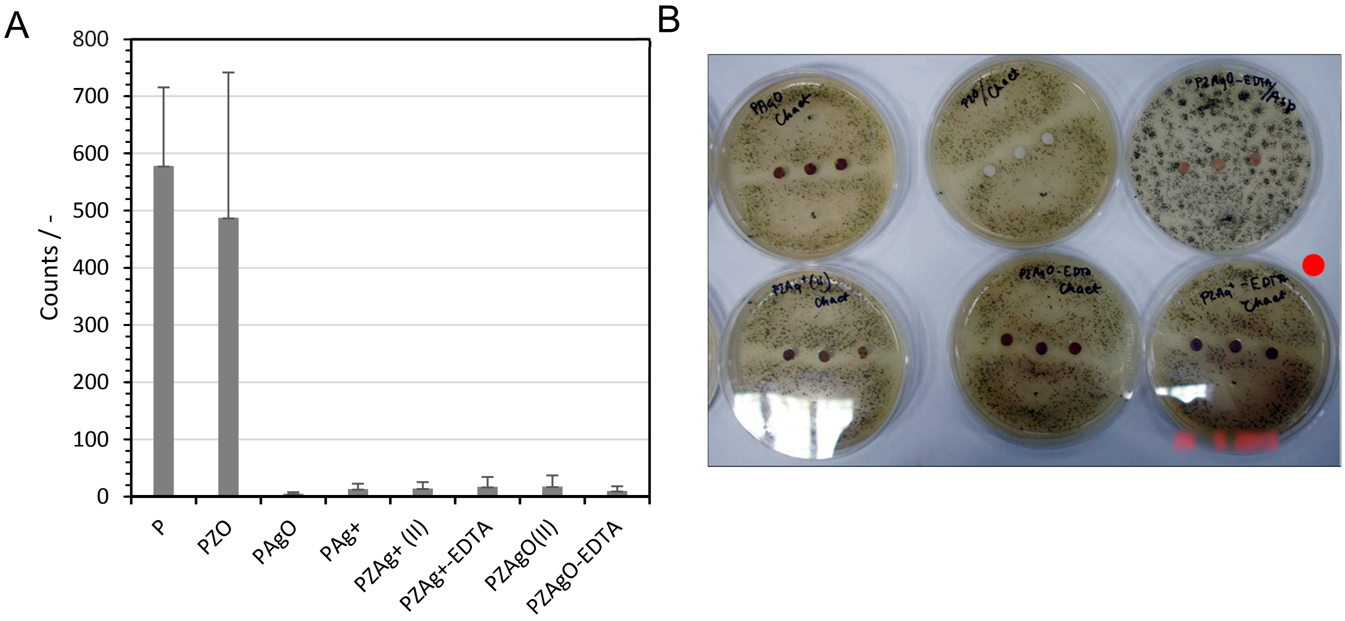

Figure S8 *Chaetomium globosum*

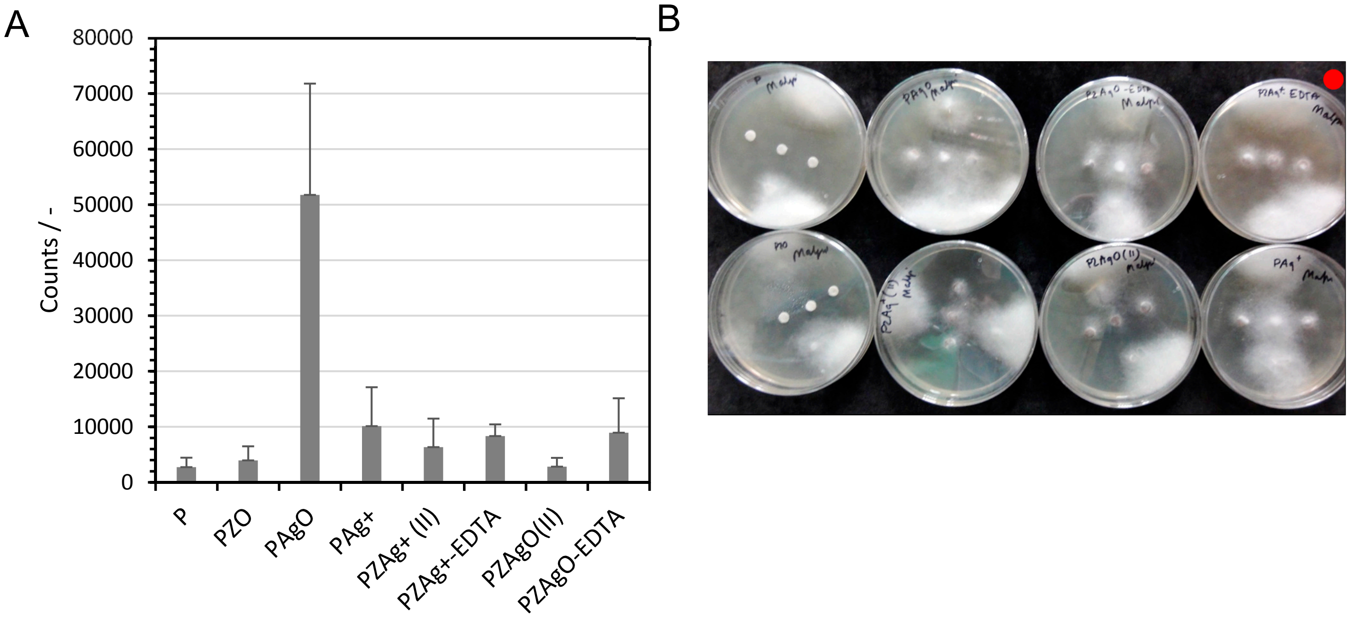

Figure S9 *Mortierella alpina*
